# Reducing relapse in children after recovery from severe acute malnutrition in Mali: participatory development of a theory of change for post-treatment monitoring including SQ-LNS supplementation

**DOI:** 10.3389/fnut.2026.1765714

**Published:** 2026-04-08

**Authors:** Suvi T. Kangas, Issa Niamanto Coulibaly, Koniba Diassana, Mamadou Zie Traore, Alhousseyni Haidara, Bernardette Cichon, Stephen R. Kodish, Grace Heymsfield

**Affiliations:** 1 International Rescue Committee, Brussels, Belgium; 2 International Rescue Committee, Bamako, Mali; 3 International Rescue Committee, London, United Kingdom; 4 Pennsylvanla State University, University Park, PA, United States; 5 International Rescue Committee, New York, NY, United States

**Keywords:** acute malnutrition, Mali, relapse, severe acute malnutrition (SAM), SQ-LNS small-quantity lipid-based nutrient supplements, theory of change (ToC)

## Abstract

**Introduction:**

Up to 76% of children treated for acute malnutrition relapse within 6 months from discharge. Despite its known negative impact on children's health and the drain on health system's scarce resources, few studies have explored ways to reduce relapse. In order to design an intervention that can work, there is a need to thoroughly understand the drivers of relapse in a context.

**Objective:**

We aimed to develop a robust theory of change (ToC) for how relapse can be reduced in Mali and propose an intervention package that is feasible for the health system to adopt with minimal external support.

**Methods:**

We applied a four-step approach to develop the ToC including: (1) An evidence review of potential drivers of malnutrition and relapse locally, based on surveys, gray literature and scientific articles, (2) Schematization of the most important immediate and underlying causes of relapse based on step 1 in a problem tree style and development of a ToC for a program that aims to address the causes, (3) Validation of the ToC with local stakeholders, and (4) Identification of facilitators, barriers and assumptions. Most work was done through on-line workshops including participants from local Ministry of Health (MoH), a local university (USTTB) and both global and local IRC nutrition staff. One in-person workshop was organized with local stakeholders from technical partners and different levels of local health authorities to finalize the ToC.

**Results:**

Three direct causes of relapse were identified: (1) Inadequate initial treatment, (2) Inadequate dietary intake post-discharge, and (3) Frequent illness episodes combined with inadequate treatment seeking. To reduce relapse post-treatment, three aims were identified: (1) Improving initial acute malnutrition treatment, (2) Improving nutritional intake after discharge, (3) Decreasing incidence of illness and increasing use of health services. The proposed intervention was built around a program for the post-treatment monitoring of children at the treatment sites including health checks and the provision of small-quantity lipid-based nutrient supplements for children. Inputs included initial and on-going training, supplies and equipment, and supervision and coordination.

**Conclusion:**

The development of a comprehensive ToC to prevent relapse allowed us to design an intervention that tackles the key perceived and evidenced drivers of relapse in the context. This ToC will underpin a process evaluation that will need to accompany an impact evaluation to determine if and how the intervention reduces relapse.

## Introduction

Wasting, which is defined as a low weight-for-height *z*-score (WHZ) or low mid-upper arm circumference (MUAC) (1), affects 43 million children under 5 years of age (U5) at any given time globally (2). Estimates suggest that nearly one third of those children, 14 million, may be severely rather than moderately wasted (2). Acute malnutrition, which combines cases with wasting to those with nutritional edema, is generally treated following World Health Organization (WHO) guidelines (1). In 2023, around 9 million children with severe acute malnutrition (SAM; defined as a WHZ <−3, MUAC <115 mm or edema) were treated (3) with more than half expected to reach recovery within a couple of months (4). However, up to 76% of children treated successfully for acute malnutrition relapse back within 6-months from initial recovery (5–7). Predictors of relapse include low anthropometry (weight, height, MUAC or the derived z-scores) at discharge from treatment (e.g., MUAC <130 vs. MUAC ≥ 130 mm) (5, 6, 8–12), frequency of morbidity following recovery (6, 12) and high level of household food insecurity (6). However, to what extent predictors are specific to relapse and not generic to wasting incidence, and what proportion is driven by context rather than individual factors remains unknown (13).

Until 2023, no global guidelines existed for preventing relapse among children recovered from SAM. In the WHO's most recently published guidelines for the prevention and treatment of wasting, cash-based transfers were recommended for preventing relapse (1). This recommendation was based on a systematic review on relapse (9) and classified as having moderate certainty evidence, given that only one study evaluated this intervention. This study was conducted in the Democratic Republic of Congo (DRC), where the provision of monthly unconditional cash transfers, each equivalent to 70% of monthly household income with a total 6-month sum of 240 usd, reduced the cumulative proportion of SAM relapse by 30%-points during 6 months post-treatment (14). While there is no benchmark for acceptable costs for preventing relapse, 240 usd seems high, particularly considering treatment of SAM has been estimated to cost on average 148 usd in West Africa (15) and 166 usd in DRC (16). In addition to questions on the cost leading to uncertain sustainability and scalability, whether similar results would be obtained in other contexts remains to be shown and would probably depend on whether the drivers of relapse are similar. The DRC study hypothesized that cash would improve food diversity and consumption thus presuming relapse is driven by lack of means to adequate foods (and not illness or other factors). In 2025 the DRC was rated as third worst country in terms of Global Hunger Index, indeed, suggesting generally poor access to food (17). The WHO has acknowledged the limited evidence base for effective relapse prevention and has called for additional research to support recommendations for post-treatment interventions (1).

A promising intervention that has been integrated into a list of effective interventions to combat child malnutrition (18) is the daily consumption of small-quantity lipid-based nutrient supplements (SQ-LNS) which provide both energy (118 kcal) and 22 micronutrients important for healthy growth and development (19). When consumed by children aged 6–23 months as a supplement to complementary foods, SQ-LNS been shown to reduce mortality (20), severe wasting (21), severe stunting (21), iron deficiency anemia (22), developmental delays (23), and wasting relapse (24).

Whether SQ-LNS provision to all children treated for SAM after recovery may be integrated into wasting treatment services remains to be explored. Compared to cash transfers, SQ-LNS can be managed through the existing health systems supply chains—similar to how ready-to-use therapeutic foods (RUTF) are procured and managed for SAM treatment—without requiring new systems for cash (in most contexts). And unlike approaches based solely on behavior change communication (BCC), SQ-LNS may provide a concrete incentive for caregivers to attend health care services (26). In the same way as RUTF, purchase of SQ-LNS could also potentially be supported by external funders which is not as easily the case for non-product based support. Having a unique target group would also help monitor use of the product, unlike the case of medicines that can be prescribed across different targets making tracking of the use of supplies provided (potentially for free) for a specific target difficult.

We recently conducted formative research to explore if and how post-treatment monitoring with SQ-LNS supplementation could be added to the SAM treatment program as an additional activity in the Kati district of Mali (26). The positive results raised further questions about how this program could be optimized to prevent relapse—namely, whether a broader approach that determines and targets all key factors underlying relapse would be more effective. To ensure interventions target the main causes of problem and that there is a clear understanding of the pathways to impact, there is a need to build a tailored theory of change (ToC) (25).

A ToC is a generally a model that depicts how an intervention is supposed to work (27). In order to build interventions that work, there is a need to design them with the specific contexts in mind (28). Building a ToC participatively (29) can also help with designing more sustainable solutions and with defining and sharing accountability (30, 31). Furthermore, ToCs are important for designing robust process evaluations of interventions to understand how a program results or fails to result in impact (32).

Here we describe the co-design of a ToC for preventing relapse in Mali through a post-treatment intervention that includes SQ-LNS supplementation of children and that is adapted to the context in terms of addressing the key local causes of relapse and proposing solutions that build on existing systems. This ToC is expected to serve as a guide for a process evaluation that will need to accompany an impact evaluation in order to understand how a reduction in relapse is achieved or why it is not achieved.

## Methods

### Setting

The ToC was designed specifically for the Kati district of the Koulikoro region of Mali. Koulikoro region is located in Western Mali and surrounds Mali's capital Bamako. It is the third largest region of the country with a population estimated at 2.3 million in 2022 (33). Koulikoro has 11 districts, the largest of which is Kati where an estimated 813,682 people and 162,763 children U5 lived in 2024 (34).

In Mali, health care is decentralized and integrated community case management (35) is used as a strategy to treat common child illnesses (e.g., malaria, pneumonia, diarrhea, and acute malnutrition) at community level by community health workers (CHWs). These paid CHWs report to community health centers (CSComs) where primary level services are available and that are managed by community health associations (ASACO). CHWs supervise unpaid volunteers (CHVs) who support health promotions during campaigns. Referral health centers (CSRef) and district hospitals provide second level of services including inpatient care and national and university hospitals represent the third level services.

In 2024, 11.6% of children U5 were estimated to suffer from acute malnutrition in Mali, similar to the 11.0% in Koulikoro region (36), both above the 10% threshold for classifying the situation as severe according to the Integrated Food Security Phase Classification (IPC) (37). The same year more than 190 000 children were treated for SAM in Mali (34), most as outpatients through CHW and CSCom levels and some as inpatients through CSRefs and hospitals in case of medical complications. SAM relapse rates are neither available nationally nor for the Kati district, but ranged from 26% (12) to 33% (6) in Nara and Kayes districts, respectively.

### Design

This work was designed to be participatory, involving four iterative steps that we adapted from USAID's suggested ToC development process for improving food and nutrition security (USAID, 2024): (1) A mapping of local evidence on the causes of relapse, (2) Development of a problem tree explaining relapse and a ToC to prevent relapse, (3) Validation and finalization of the problem tree and ToC, and (4) Identification of facilitators, barriers and assumptions for the ToC to work.

#### Step one: evidence generation

This step consisted of mapping existing evidence on factors influencing health and nutritional status in Mali and specifically the Koulikoro region and Kati health district based on UNICEF's conceptual framework on malnutrition (38). Contextual evidence was derived from numerous sources, including Demographic and Health Surveys (DHS) (39, 40), Standardized Monitoring and Assessment of Relief and Transitions (SMART) surveys (36), Semi-quantitative Evaluation of Access and Coverage (SQUEAC) surveys (41), local health system information reports (42), qualitative investigation reports (43), Nutrition and Food Security reports (44, 45), published literature (6, 12) and other relevant local sources (34). Evidence was compiled in an Excel sheet where the available data points on the evidence for each determinant were noted including the source, the year and the administrative level of representativity.

#### Step two: ToC development

This step involved developing a problem tree and a ToC based on available evidence and local knowledge. Participants first worked in teams to identify and reach consensus on the direct causes of relapse in the local context. Teams were composed to maximize heterogeneity in employer and global vs. local perspective in each group. Each group then worked to identify the underlying causes behind one direct cause. Once a consensus was reached on the problem tree, groups translated the causes into outcomes and added outputs, processes and inputs into the ToC. The inputs were developed with expected cost and feasibility in mind aiming to develop a package that builds on and can be easily incorporated into the national nutrition treatment protocol and wouldn't necessarily require NGO support or multi-actor coordination for implementation. However, no limitations were provided with regards to inputs targeting more structural (underlying) vs. direct causes of relapse. The ToC also drew on the idea for a post-treatment monitoring program that would provide SQ-LNS supplementation to children after discharge from acute malnutrition treatment (26). Miro software was used as a tool to co-develop the problem tree and the ToC (Miro, Inc., San Francisco, California, US).

#### Step three: stakeholder validation

This step involved a participatory workshop to revise the problem tree and ToC with a larger audience of local stakeholders in an interest to obtain relevant inputs and finalize and validate the content. Participants were purposively selected to represent different levels of the health care system (community, health center, district, regional, central) and different angles (health system, technical partners, researchers etc). Participants were divided into three groups, each tasked with critically reviewing the problem tree and the theory of change, and working separately on one of the three pathways. Pre-printed components were sketched to flip charts to present the ToC and add any additional components with the help of sticker notes.

#### Step four: identification of facilitators, barriers and assumptions

This step involved a systematic and critical appraisal of the links between inputs and processes, processes and outputs, outputs and outcomes, and outcomes and impact to identify factors that facilitate (*Facilitators*) or hinder (*Barriers*) progress along each pathway. *Assumptions* were defined as both conditions that are in place and that we expect to continue being in place regardless of the project and any causal connections underlying the links in the impact pathway. Miro software was used to guide the reflection and comprehensively identify facilitators, barriers and assumptions between each step from inputs to impact.

### Implementation

Steps one, two and four were conducted through on-line workshops and step three was conducted through an in-person workshop held in Bamako (Table 1). Not all 10 participants in on-line workshops were available at all 13 workshops. A mean of seven participants was recorded throughout on-line sessions.

**Table 1:** Description of the theory of change workshop organization and attendees.

Aspects	On-line workshops	On-site workshop
Steps conducted	1, 2, 4	3
Number of sessions	13	1
Duration of sessions	1 h 30 min	3 h
Number of attendees (participants + facilitators)	10 + 1	16 + 6
Occupation of attendees	Nine IRC staff, 1 MoH staff, 1 USTTB researcher	One WFP staff, one Mali Nutrition Cluster staff, two MoH staff, one regional health authority, two district health authority, one district administrative authority, three technical partners, three health care workers (each from different facility), one health researcher, two community health platform member (each from different community)
Number of attendees with global vs. national expertise	Seven national experts, Four global experts	22 national experts

### Ethics

The International Rescue Committee Internal Review Board (OHRP# IORG0008134) determined the study exempt from full review and waived the need for informed consent as this was deemed as a research activity. Participants were recruited on a voluntary basis.

## Results

**Figure 1:**
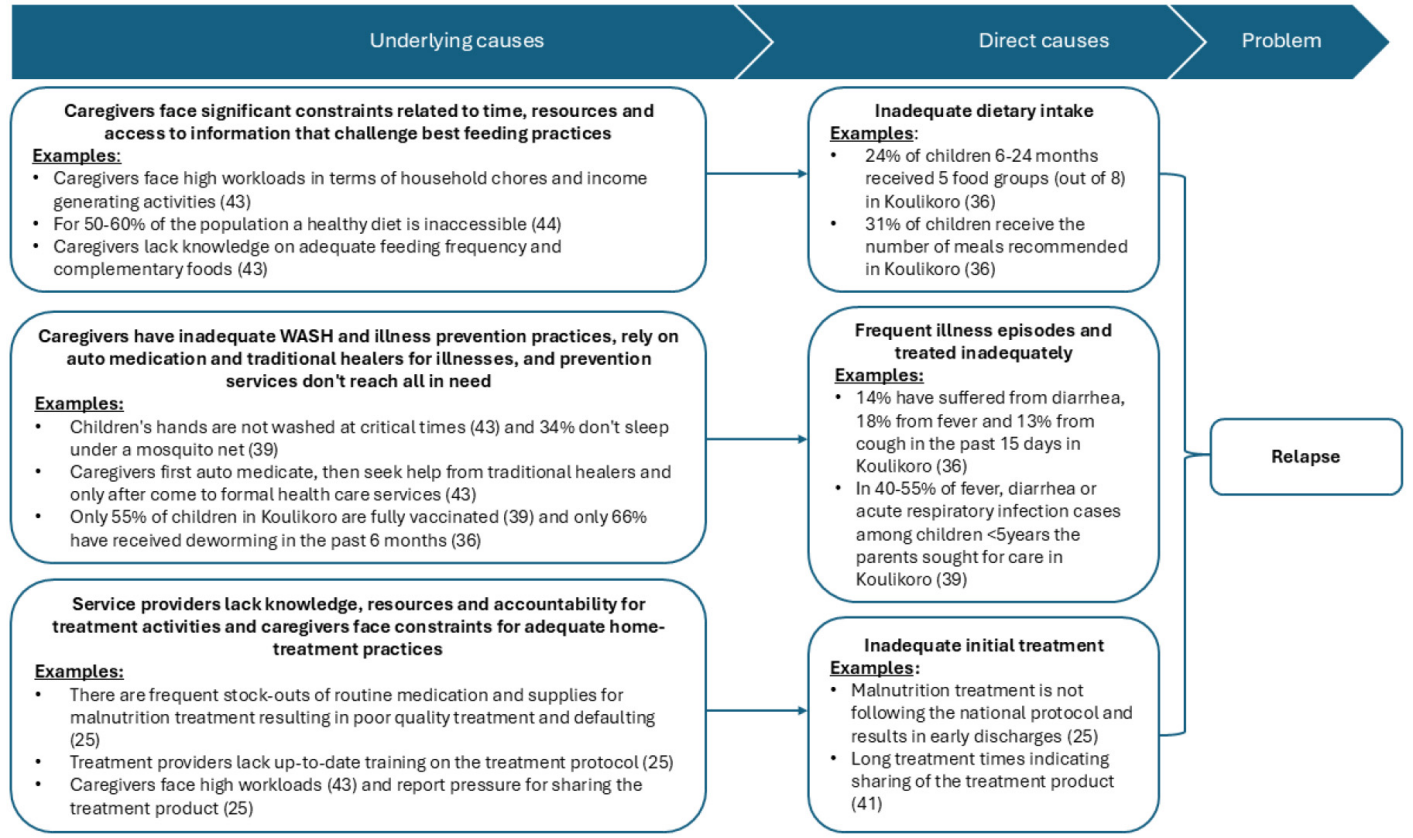
Illustration of direct and underlying causes of relapse in Mali and key evidence supporting these causes.

**Figure 2:**
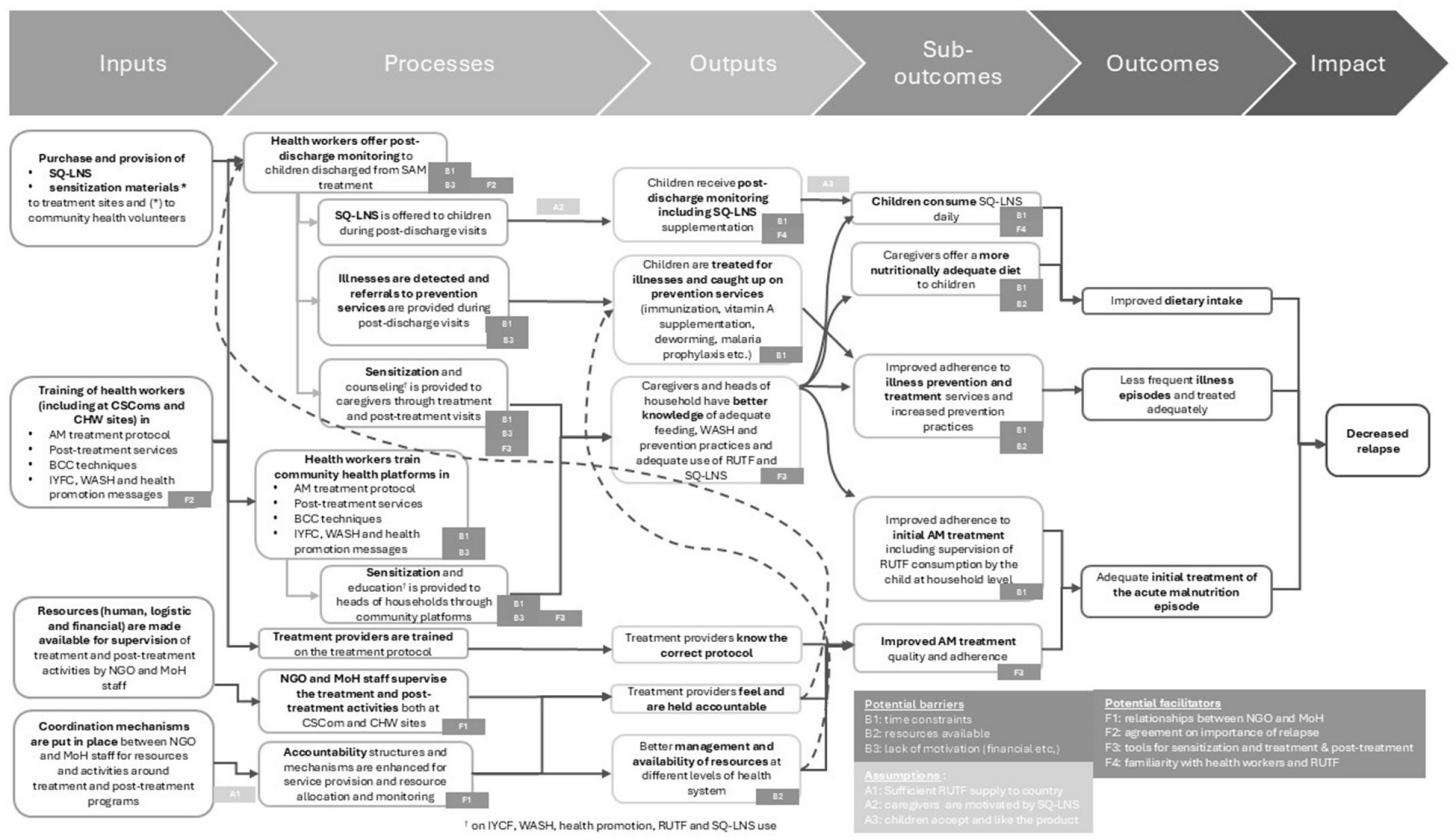
Theory of Change for reducing relapse in the Kati district of Mali through a program proposing post-treatment monitoring of children after treatment and providing on-going training and regular supervision of health staff. AM, acute malnutrition; BCC, behavior change communication; CHW, community health worker; CSCom, Center de Sante Communautaire = Community Health center; IYCF, infant and young child feeding; NGO, non-governmental organization; MoH, Ministry of Health; RUTF, ready-to-use therapeutic food; SQ-LNS small-quantity lipid-based nutrient supplement; WASH, water sanitation and hygiene.

Based on the problem tree (Figure 1) that was constructed with the help of the evidence map (Supplementary Table 1), the ToC was developed to target the three direct causes that were seen to drive relapse: (1) Inadequate dietary intake, (2) Frequent illnesses combined with infrequent care seeking and (3) inadequate initial treatment of the malnutrition episode (Figure 2). This ToC comprised of a comprehensive program including support to the treatment program and integrating a post-treatment monitoring component for children after treatment and that includes SQ-LNS supplementation along with routine medical checks, regular growth monitoring, health promotion, and timely treatment of common illnesses and referrals to preventive services. The program included three key input types: (1) provision of SQ-LNS to the treatment sites, (2) initial training of the health workers in the treatment protocol, the post-treatment monitoring activity as well as key health promotion and illness prevention messages to provide to caregivers and (3) regular coordination and joint supervision of activities by the NGO and health authorities at regional and district level (Figure 2).

The ToC suggested that SQ-LNS will (1) help children reach adequate nutritional intakes by providing them a highly nutritious supplement (SQ-LNS) and by improving meal frequency and intake of other highly nutritious foods via sensitization of caregivers and husbands and (2) act as an incentive to the caregivers to attend regular monitoring visits during which common illnesses can be detected and treated and routine preventive measures (vitamin A supplementation, deworming, vaccination) ensured and advice given to prevent common illness such as malaria, diarrhea, respiratory infections etc. In addition, the coordination, training and supervision of activities will improve initial treatment implementation and the quality and consistency of health services provided. Training was planned to be incorporated into current CMAM training curricula and to include specific modules on post-treatment services (including anthropometric checks, clinical exams, counseling and distribution of SQ-LNS), in addition to the already existing initial treatment, BCC techniques and IYCF, WASH and health promotion content (46).

Potential facilitators for different components of the program included (but were not limited to) (1) good relationships between different actors including between IRC and the district, regional and central health authorities as well as with health workers and population, and between health workers and community health platforms, (2) alignment between actors including central, regional and district health authorities, health care workers and population on the importance of preventing relapse, (3) context adapted tools and guidance for health promotion, treatment program and post-treatment program, and (4) familiarity of caregivers with the treatment program including the health workers and RUTF.

Potential barriers for different components of the program included (but were not limited to) (1) time constraints experienced by both the caregivers to participate in program and ensure their child consumes SQ-LNS daily, and health workers to provide adequate services and BCC messaging, (2) resource constraints experienced both by the population and leading to inability to act upon some BCC messaging and by the health system leading to inability to ensure adequate supplies for treatment and prevention services, and (3) lack of additional payments to health workers, CHWs, CHVs etc. leading to lack of motivation to provide services appropriately and to perform health promotion.

Assumptions underlying the success of the relapse prevention program included that (1) the RUTF and routine SAM medication pipeline for the country remains secured, (2) caregivers are motivated by the SQ-LNS to attend post-treatment monitoring visits, and (3) children like and accept the SQ-LNS product.

## Discussion

This study aimed to describe the process used to build a robust ToC to prevent relapse in Mali. We first developed a problem tree explaining relapse in Mali, which informed the design of an intervention package that targets key causal factors of relapse aiming at (1) improving nutritional intake of children, (2) decreasing incidence of illness and increasing use of health services and (3) Improving initial acute malnutrition treatment. The intervention was built on the idea of a post-treatment monitoring program that includes SQ-LNS supplementation of children (26). Key inputs included (1) training of health care staff and community health volunteers, (2) provision of SQ-LNS to treatment sites (3) supportive supervision of health workers and coordination and oversight of activities.

The key drivers considered to cause relapse represent the two causal paths of the UNICEF framework for malnutrition (38) that suggests diet and illnesses as the most immediate causes of malnutrition, and are complemented by the inclusion of initial treatment quality as per the more recent conceptual framework developed for relapse (13). These also reflect recent evidence on relapse identifying severe food insecurity (6) and illness episodes (6, 12) during post-treatment period and low anthropometry at discharge (6, 12) as predicting relapse in Mali. Globally, few common risk factors have been identified (6, 13) which speaks to the need for considering local evidence in understanding the drivers of relapse before the design of interventions to tackle relapse. Also, a recent meta-analysis indicates that anthropometric recovery may precede immunological recovery suggesting that children would benefit from health monitoring post-treatment (5).

While the Mali national protocol for management of acute malnutrition includes a post-treatment monitoring phase for children discharged as recovered (47), this has been shown not to be implemented in practice due to lack of training of health care workers in this activity (26). However, this same work suggested that these monitoring visits would be feasible for the health system to put in place and desirable for the caregivers (26). Thus, our ToC builds on this opportunity to provide post-treatment visits and sought to understand what services these visits should offer and what kind of support the health system would need to successfully implement such a program.

The inputs proposed to reduce relapse (e.g., training, nutritional product, supervision and coordination) are aligned with key enablers identified for effective provision of malnutrition treatment services in general (48). Training has been identified as critical for ensuring SAM treatment provision in Ghana (49) and supervision has been shown crucial to achieving acceptable quality of SAM treatment in Mali (50). SQ-LNS has been shown to motivate caregivers to attend meetings organized by CHVs at village level in Mali (51) or by health workers at the health care site in Burkina Faso (52) to screen and provide behavior change communication messaging to caregivers of children 6–23 months of age. Evidence has also shown that educational interventions can improve dietary (53) and illness prevention (54) practices in developing countries. Thus, while driven by local level insights, the inputs are backed by global evidence and can reasonably be expected to result in improved initial treatment, improved dietary intakes and reduced morbidities in this setting.

The proposed intervention targets mainly the direct drivers of relapse and not the underlying causes (38). Nutritional supplementation combined with post-treatment monitoring provides nutrients while creating regular contact with health services to detect and treat illness early. Thus, the intervention operates through a more direct biological and clinical pathway as opposed to more structural pathways that often depend on more distal pathways. Cash assistance for example is generally aimed at addressing poverty and household purchasing power and thus assumes that these are the main underlying drivers of inadequate behaviors. From a health-system approach perspective, direct interventions may be more feasible than structural ones though more research would be needed to compare both feasibility and effectiveness.

There are many ways a ToC can be developed and presented (27), often depending on the use case (55): whether to help design an intervention package or rather to evaluate the effectiveness of one. In our case, while the core intervention concept was defined (post-treatment monitoring with SQ-LNS supplementation), we sought to maximize its potential impact by designing supportive components that address other key factors contributing to relapse. This required a thorough mapping of the direct and underlying expected causes of relapse and the subsequent identification of activities that can target these challenges. We believe that the four-step method was well-aligned with our needs and resulted in a ToC that is clear, grounded in local evidence and insights and accessible to key stakeholders including front line implementors.

The proposed post-treatment monitoring program with SQ-LNS supplementation will next be tested for feasibility and impact on relapse. Implementation fidelity may be improved by programming that addresses the barriers of time constraints, resource constraints, and lack of health worker payments which were identified as potential bottlenecks. This ToC will also help with building a theory-based impact evaluation (56) that will allow for a more nuanced understanding of both the extent to which, and how (25), the program works to reduce relapse risk.

To our knowledge, this is the first time that the development of a ToC to prevent relapse in a specific setting has been reported in scientific literature. Generally, the acute malnutrition sector as a whole has suffered from a lack of well-described impact pathways for interventions being tested and from a “magic bullet” thinking whereby a single intervention is expected to result in major impacts (57, 58). Where theories of change have been described (51, 52, 59), they are typically part of a study protocol description, lacking details on who was involved in the development, as well as whether and how context specific causes of malnutrition were considered in the development process. Not having a well-defined theory of change that is based on the context where implementation is taking place can lead to interventions that are not addressing the main causes of the problem and thus may turn out ineffective (60). Involving local stakeholders in the development process is important, not only for identifying the local causes of the problem but also for planning interventions that can be integrated into existing systems and scaled if shown effective (61). Overall, there is a call for more implementation science in health (62) and nutrition specifically (63), including in designing (and reporting the design process of) more robust interventions that can work at scale.

The ToC was developed principally through on-line workshops with local stakeholders, including NGO staff working in nutrition research and programs and one health ministry official and one national level researcher. Only a half-day validation exercise was led with a broader local stakeholder group including implementors and community representatives. This validation exercise proved useful as there were relevant additions made to the problem tree and theory of change by the stakeholders. Further involvement of an even broader group of stakeholders including service users could have provided further validation and potential additions to the ToC. Future endeavors to develop a ToC are encouraged to reserve a full day for a theory of change validation exercise and to include potentially a separate step to involve service users as well.

Given the context-specific evidence and insights used to develop the intervention, the ToC may not be directly applicable to other contexts and programs. However, we hope that the detailed description of the ToC development process will inspire other researchers to both invest in and document this step that simultaneously allows others to understand the reasoning behind a specific intervention design and to consequently adapt it to their own context.

### Strengths

A key strength of this study was the thoroughness of the local evidence mapping that helped the research team to determine the key problems underlying relapse. This also helped reduce individuals bias toward specific determinants as justification was based on the evidence map and consensus was sought for the final version of the problem tree. The participatory nature of the theory development also ensured that local perspectives were integrated into the ToC and the purposive sampling ensured diversity in perspectives. In addition, parallel work by the study team on formative research for a post-treatment program including SQ-LNS supplementation (26) strengthened the teams understanding of the local context and provided good insights into potential gaps and needs.

### Limitations

This study also has some limitations. First, due to resource constraints, we could not organize community consultations on the ToC and thus did not validate the final version with the community. Doing so may have strengthened our confidence in the proposed approach for preventing relapse by ensuring beneficiary perspectives were accounted. Second, our ToC was not totally open ended in terms of imagining what is needed for reducing relapse but built on the idea of a post-treatment monitoring program with SQ-LNS supplementation (26). While the program approach was somewhat given, the supportive components (trainings, behavior change communication, supervision and coordination) were designed and fine-tuned based on the problem analysis. Without the initial orientation, we would expect post-treatment monitoring to still be key to reducing relapse but potentially, instead of SQ-LNS, some other products or services might be proposed.

## Conclusion

Using a participatory process among diverse stakeholders was an informative and effective method for developing a tailored ToC that helped guide the design of a post-treatment monitoring programming for children discharged from acute malnutrition treatment in Mali. The program included training, provision of SQ-LNS, supportive supervision and coordination and oversight of activities. These actions were defined as key to improving nutritional intake of children, decrease incidence of illness, increase the use of health services and improve initial acute malnutrition treatment. Actors in other contexts considering tackling the problem of relapse may follow a similar process that considers the local evidence and stakeholder insights on drivers of relapse and on approaches needed to tackle it.

## Data Availability

The original contributions presented in the study are included in the article/Supplementary material, further inquiries can be directed to the corresponding author.
