# Correction: Reducing relapse in children after recovery from severe acute malnutrition in Mali: participatory development of a theory of change for post-treatment monitoring including SQ-LNS supplementation

**DOI:** 10.3389/fnut.2026.1863256

**Published:** 2026-05-12

**Authors:** Suvi T. Kangas, Issa Niamanto Coulibaly, Koniba Diassana, Mamadou Zie Traore, Alhousseyni Haidara, Bernardette Cichon, Stephen R. Kodish, Grace Heymsfield

**Affiliations:** 1 International Rescue Committee, Brussels, Belgium; 2 International Rescue Committee, Bamako, Mali; 3 International Rescue Committee, London, United Kingdom; 4 Pennsylvanla State University, University Park, PA, United States; 5 International Rescue Committee, New York, NY, United States

**Keywords:** acute malnutrition, Mali, relapse, severe acute malnutrition (SAM), SQ-LNS small-quantity lipid-based nutrient supplements, theory of change (ToC)

In Methods, **Ethics statement** was erroneously given as “The International Rescue Committee Internal Review Board (OHRP# IORG0008134) determined the study exempt from full review. Participants were recruited on a voluntary basis. For research inclusivity purposes (46) and given that all work was conducted initially in French, a French version of the manuscript is provided as Supplementary File 1”.

The correct **Ethics statement** is: “The International Rescue Committee Internal Review Board (OHRP# IORG0008134) determined the study exempt from full review and waived the need for informed consent as this was deemed as a research activity. Participants were recruited on a voluntary basis. “

In addition, the reference number 46 “Roca A, Boum Y, Wachsmuth I. Plaidoyer contre l'exclusion des francophones dans la recherche en santé mondiale. *Lancet Glob Health*. (2019) 7:e701–2. French. doi: 10.1016/S2214-109X(19)30175-5” should be removed and citation numbers and list updated.

The original version of this article has been updated.

