# Supplementary material for: Reducing relapse in children after recovery from severe acute malnutrition in Mali: participatory development of a theory of change for post-treatment monitoring including SQ-LNS supplementation
Source: Front Nutr. 2026 Apr 8;13:1765714. doi: 10.3389/fnut.2026.1765714 (PMC13099773; doi:10.3389/fnut.2026.1765714)
Supplement: Supplementary file 1 [file Table_1.docx]

Supplementary Table 1: Evidence against potential causes of malnutrition and relapse according to UNICEF conceptual model (36)

| **Category** Sub-category | **Topic** | **Evidence** | **Administrative area** | **Reference** |
| --- | --- | --- | --- | --- |
| **Manifestation** |  |  |  |  |
| Malnutrition & relapse | Acute malnutrition | 11% GAM by WHZ<-2 (average in Mali = 12%) | Koulikoro | 36 |
|  |  | 5.5% WHZ<-2 (average in Mali = 5.4%) | Koulikoro | 39 |
|  |  | 47 000 SAM admissions in 2022 (3rd largest after Mopti=71 000 and Gao=51 000) | Koulikoro | 42 |
|  |  | 1300 SAM admissions in 2024 | Kati | 34 |
|  | relapse | 26% of children with MUAC<115 treated relapsed into MUAC<125mm within 6-months | Nara | 12 |
|  |  | 30% of children with MUAC<115mm or WHZ<-3 relapsed to GAM by 6-months (7% to SAM) | Kayes | 6 |
| **Immediate causes** |  | | | |
| Diet | Diet diversity | 24% of children 6-24 months old received 5 food groups (out of 8) (national average= 27%) | Koulikoro | 36 |
|  | Diet frequency | ​31% of children receive the number of meals recommended (national average = 53%) | Koulikoro | 36 |
|  | Unhealthy diets | 36% of children <2years consume sugar sweetened beverages | Mali | 39 |
|  |  | 29% of children <2ans consume unhealthy foods | Mali | 39 |
|  | Breastfeeding | 97% of children 6-12 months are still breastfed (national average = 97%) | Koulikoro | 36 |
|  |  | 47% of children 12-24m are still breastfed (national average =50%) | Koulikoro | 36 |
| Care practices | Careseeking | ​In 40-55% of fever, diarrhea or ARI cases among children <5years the parents sought for care (national average similar) | Koulikoro | 39 |
|  |  | in case of illnesses, caregivers first use auto medication, then seek help from a traditional healer and only thereafter go for formal care at the health center | Kati | 41 |
|  | Accessibility | Distance from care combined with lack of means of transportation are one barrier to access to malnutrition treatment | Kati | 41 |
|  |  | 10% of the population lives +15km of health services (national average = 11%) | Koulikoro | 42 |
|  | Challenges | "malnutrition doesn't seem so much related to the economic situation but rather to the resource allocation and decision making power within households and to the negligence of children (who stayed long moments without eating) and to the lack of knowledge on optimal feeding practices" | Yorosso and Bankass | 43 |
|  |  | "hygiene practices seem really poor, the children's hands are never washed before meals etc" | Yorosso and Bankass | 43 |
|  |  | fathers and grand mothers seemed key in decision making for what can be purchaised | Yorosso and Bankass | 43 |
|  |  | health agents didn't give a lot of information et weren't the 1st source of information to the caregivers (rather older women were) | Yorosso and Bankass | 43 |
|  | Prevention | 60% of children 6-59m have been dewormed in the past 6months (national average = 77%) | Koulikoro | 36 |
|  |  | 90% of children 6-59m have received vitamin A in the past 6months (national average = 86%) | Koulikoro | 36 |
|  |  | 73% of children <5years have a mosquito net in their household (national average = 73%) | Koulikoro | 39 |
|  |  | 66% of children <5years sleep below a mosquito net (national average = 67%) | Koulikoro | 39 |
|  | Illnesses | 14% have suffered from diarrhea in the past 15 days (national average = 12%) | Koulikoro | 36 |
|  |  | 18% had fever/malaria in the past 15 days (national average = 18%) | Koulikoro | 36 |
|  |  | 13% had cough in the pasts 15 days (national average = 14%) | Koulikoro | 36 |
|  | Mortality | mortality rate = 52/1000 children <5 years | Koulikoro | 39 |
|  |  | severe malaria represents 80%of case fatality in children <5ans (followed by accidents, ARI and anemia) | Mali | 42 |
|  |  | the incidence of malaria among children <5 years of age is higher than the national average | Koulikoro | 42 |
|  |  | the mortality rate due to malaria is highest in all country (incidence=136, national average=77) | Koulikoro | 42 |
|  | Vaccination | vaccination coverage (number of doses distributed/target population) >100% for all vaccines (except var2) (national average = 90-100%) | Koulikoro | 42 |
|  |  | 55% des enfants <2ans are completely vaccinated (national average = 52%) | Koulikoro | 39 |
|  |  | 10% of children <2years hadn't received any vaccin(national average = 13%) | Koulikoro | 39 |
|  | Health care offer | 5 health care professionals/10 000 habitants (national average = 5, WHO recommendation = 23) | Koulikoro | 42 |
| Initial treatment | Caregiver practices | Treatment duration is long indicating inadequate home-treatment practices by caregivers (sharing of RUTF ration) and by health care agents | Kati | 41 |
| **Underlying causes** |  | | | |
| Food | Accessibility | 66% of expenses of the households go to food (national average=63%) | Koulikoro | 45 |
|  |  | insecurity is the main reason for limited accessibility to market (but access is relatively good and stable compared to other regions) | Koulikoro | 45 |
|  |  | for 50-60% of the population in this area, nutritional diet is not affordable | Koulikoro | 44 |
|  | Food security | 3.3% declare feeling moderate food insecurity (food security experience score) (national average = 9.9%) | Koulikoro | 45 |
|  |  | 1.1% of households in Kati have resorted to non-food strategies to cope with a crisis (national average=14%) | Kati | 45 |
|  |  | 99% of households declare not suffering from hunger (national average=97%) | Koulikoro | 45 |
|  |  | 18% have received assistance (national average=35%) (most often these are food rations or medicine) | Koulikoro | 45 |
|  |  | 47% are considered food secure (national average=35%) | Koulikoro | 45 |
|  |  | a worsening level of food security is forecasted (more than the national average) | Koulikoro | 45 |
|  | Perceptions | the quality of food and ingredients was not a value that was considered when buying and preparing foods rather the price was more important | Yorosso and Bankass | 43 |
|  |  | Two preferred cereals=millet (67%), rice(54%), sorghum (34%) | Koulikoro | 45 |
|  |  | Two preferred vegetables = onion (85%), green leaves (68%), tomatos (36%) | Koulikoro | 45 |
|  |  | Two preferred legumes = peanut (98%) et cowpea (98%) | Koulikoro | 45 |
|  |  | Two preferred roots = potato (75%), igname (66%), sweet potato (48%) | Koulikoro | 45 |
|  | Nutritional value | 83% of households have an acceptable dietary consumption score (national average = 70%) | Koulikoro | 45 |
|  |  | 47% consume vitamin A rich foods every day (national average=39%) | Koulikoro | 45 |
|  |  | 74% consume protein sources every day (national average= 66%) | Koulikoro | 45 |
|  |  | 80% consume products rich in iron (national average=70%) | Koulikoro | 45 |
|  |  | Least consumed food groups are fruits (19%), eggs (38%), tubers (37%) | Koulikoro | 45 |
|  | Water | 30% use a non-improved water source for drinking water (national average =30%) | Koulikoro | 39 |
| Practices | IYCF | 24% of children 6-24 months receive 5 food groups (national average = 27%) | Koulikoro | 36 |
|  |  | 31% of children receive the number of meals recommended (national average = 53%) | Koulikoro | 36 |
|  |  | 36% of children <2years consumed sugar sweetened beverages | Mali | 39 |
|  |  | 29% des enfants de <2ans consomment des aliments malsains | Mali | 39 |
|  | Priority | men are served first and benefit from the best parts of the meal, thereafter elderly women and only then women with young children" | Yorosso and Bankass | 43 |
|  |  | the quantity of food doesn't change by season but the quality may be adapted (more porrige than to during lean season) | Yorosso and Bankass | 43 |
|  | WASH | "children's hands are never washed before meals and women don’t wash their hands appropriately upon critical moments" | Yorosso and Bankass | 43 |
|  |  | 71% of households have a dedicated place to wash hands but 57% of these places don't have either water or soap | Koulikoro | 40 |
|  |  | 14% treat waster correctly before use in Mali | Koulikoro |  |
|  | Preparation | children eat the family meal very early and receive snacks between meals but which can be of very low quality" | Yorosso and Bankass | 43 |
| Services | CMAM | There are 44 health centers offering acute malnutrition treatment services in Kati, CHW sites have not been trained and equipped in treatment yet | Kati | 34 |
|  |  | The national protocol is not respected (RUTF dosage, exit criteria) | Kati | 25 |
|  |  | Frequent stock-outs in treatment supplies, including RUTF but particularly amoxicillin and deworming | Kati | 25 |
|  | Health and hygiene | 5 health care professionals per 10 000 habitants (national average = 5, WHO recommendation = 23) | Koulikoro | 42 |
|  |  | 10% of the population lives +15km from basic health services des (national average = 11%) | Koulikoro | 42 |
|  |  | 270 CSComs which makes it 3rd largest health area (with Kayes and Sikasso) with the largest number of CHW sites (n=743) | Koulikoro | 42 |
|  |  | 13% of population didn’t have access to water during 1 day in the 2 last weeks | Mali | 40 |
|  | Careseeking | mothers consider elderly women as the primary source of information for health and nutrition (not health care professionals) | Yorosso and Bankass | 43 |
|  |  | every village has a CHVs who knows the health&nutrition recommendations but they are not motivated to pass on the messages since they are not paid for it | Yorosso and Bankass | 43 |
| **Basic causes** |  | | | |
| Resources | Cost of diet | 66% of household expenses go to food (national average=63%, acceptable level <50%) | Koulikoro | 45 |
|  |  | for 50-60% of the population in this area nutritional diet is not affordable | Koulikoro | 44 |
|  | Poverty | 37% of the population is classified as poor or very poor (national average = 20%) | Koulikoro | 45 |
|  |  | Median income for households/month = 150k cfa (national average =130k cfa) | Koulikoro | 45 |
|  |  | Minimum necessary income /month =100k cfa (national average =100k cfa) | Koulikoro | 45 |
|  | Access to food and water | The region doesn’t experience as much seasonal difficulty in accessing foods than other regions (slight increase between June and October) | Koulikoro | 45 |
|  |  | 71% of the population uses improved sources for drinking water | Koulikoro | 40 |
|  |  | 95% of the population has <30min access to water | Mali | 40 |
|  |  | 7% has a limited access to water | Mali | 40 |
| Norms | Food | boys (starting from 3 years of age) eat with men who have a better access to food than women and girls and young boys < 3years of age | Yorosso and Bankass | 43 |
|  |  | men are in charge of resources and do not understand the importance of diet quality for the health and growth of children | Yorosso and Bankass | 43 |
| Governance | Accountability | 99% completeness of epidemiologic monitoring reports (national average=90%) | Koulikoro | 42 |
|  |  | 55% timeliness of epidemiological monitoring reports (national average = 52%) | Koulikoro | 42 |
|  | Policy and protocols | CMAM protocol is reviewed and updated regularly and treatment by CHWs is included in the minimum package of activities | Kati | 25 |
|  |  | Health agents have not received training in CMAM for +10ans | Kati | 25 |
|  | Stability | 25% of households declare having faced a shock that had a negative impact on their access to food (national average= 38%) | Koulikoro | 45 |
|  |  | insecurity is the main reason for challenges in accessing the market in Koulikoro (but it remains relatively stable and good compared to other regions) | Koulikoro | 45 |
